# Aesthetic surgery complications in private practice settings: Insights to optimize patient access and outcomes

**DOI:** 10.1016/j.jpra.2026.05.052

**Published:** 2026-06-06

**Authors:** Nina Yu, Scott Levin, Granger Wong

**Affiliations:** Division of Plastic and Reconstructive Surgery, University of California Davis Medical Center, Sacramento, CA, United States

**Keywords:** Aesthetic surgery, Cosmetic tourism, Health equity

## Abstract

**Background:**

Complications of aesthetic surgery performed in private practice pose significant risk to patients and resource burden to healthcare systems. Thorough understanding of this population is important to mitigate future complications. We evaluated demographic and complication patterns among patients presenting to our institution after aesthetic surgery, and the financial liability of their care on the hospital and payor.

**Methods:**

This was a retrospective chart review of patients with complications related to aesthetic procedures from 2020–2023. Extracted data include biologic sex, age, smoking status, geographic location of original procedure, index surgical complications, timing of presentation, imaging, length of hospital admission, hospital charges, and insurance payments. Univariable analyses identified associations with outcomes.

**Results:**

A total 36 patients were included (mean age: 37.5 years). Patients were more likely to be Black (p = 0.04), Hispanic (p = 0.03), and on Medicaid (p < 0.001). Tobacco/cannabis use and obesity were prevalent in 25% and 47.2% of patients, respectively. Nearly half of patients had their procedures in Mexico (36.1%) and the Dominican Republic (11.4%). Patients using tobacco/cannabis were more likely to present with infection (p = 0.001) and undergo intervention (p = 0.01). The total median hospital charge was $43,324.96 (IQR $10,728.12, $80,803.18) and median insurance payment was $3947 (IQR $404.61, $24,516.00). In cases involving operative intervention, median hospital charge and insurance payments were $125,358.70 (IQR $51,065.52, $152,704.70) and $14,863.52 (IQR $3947, $49,031.13), respectively.

**Conclusion:**

There is a disproportionate number of patients from socioeconomically disadvantaged backgrounds who present with aesthetic complications and incur substantial costs, prompting the need for interventions that promote safe, equitable access to aesthetic procedures.

## Introduction

### Background

Elective cosmetic procedures are burgeoning in demand, with patients seeking care at private facilities in the United States and abroad.^1^, ^2^, ^3^ This trend has been met with offerings from some private facilities that initially attract patients with lower procedure costs and/or vacation style amenities, but the complications that arise from these procedures generate resource burdens on patients, providers, and healthcare systems alike.

For patients, studies show that not only can they face questionable surgical practices such as silicone as a proxy filler for augmentation,^4^ but the complications can be life-threatening, with case reports of death.^5^ Additionally, studies have demonstrated that these patients present to and are managed by plastic surgeons who have not been involved in their index surgery.^6^^,^^7^ There remains limited understanding of the demographic characteristics of patients presenting with these complications, particularly regarding socioeconomic status and insurance coverage. Furthermore, the specific risk factors associated with complications and the financial burden on public healthcare systems require further elucidation.

### Objectives

This study aims to evaluate demographic and complication patterns among patients presenting to an academic center after aesthetic surgery performed at outside facilities, and to quantify the financial and resource burden of their postoperative care on the payor and hospital.

## Methods

We performed a retrospective chart review of patients who presented to our institution from 2020–2023. Inclusion criteria were patients with primary complaints related to aesthetic procedures performed at outside centers resulting in a plastic surgery consultation. Patients were identified through Epic electronic medical record review using relevant diagnostic and procedural codes. Approval for this study was obtained by the institutional review board (IRB study #1593453-2). We followed the Strengthening the Reporting of Observation Studies in Epidemiology (STROBE) guidelines for reporting observational cohort studies.

### Data collection

Extracted data included biologic sex, age, race/ethnicity, smoking status (i.e. tobacco and cannabis use), body mass index (BMI), geographic location of original procedure, index surgery and respective complication(s), timing of presentation to our institution, imaging studies performed, length of hospital admission, whether intervention was conducted at our institution and if so, the type of intervention performed, hospital charges, and insurance payments. Obesity was defined as BMI ≥30 kg/m², in accordance with the World Health Organization guidelines.^8^ Those with missing data were excluded from analysis.

### Financial analysis

Hospital charges and insurance payments per admission were obtained. Total costs included the index hospital encounter and any subsequent outpatient or emergency department visits related to the complication. Costs were analyzed separately for patients who required operative intervention versus those managed conservatively.

### Statistical analysis

Demographic comparisons were performed against the overall Emergency Department population during the study period to contextualize disparities in healthcare utilization and resource burden associated with patients presenting with complications after aesthetic surgery performed at outside institutions. Within the study cohort, univariable analyses were performed to identify associations between patient characteristics and clinical outcomes. Statistical significance was defined as p ≤ 0.05.

## Results

### Patient demographics and characteristics

A total 36 patients were included with demographics enumerated in Table 1. Mean age was 37.5 ± 9.8 years. All patients were female. Compared with the rest of the patient population presenting to the Emergency Department over the same time period (n = 269,286), the identified patients were more likely to be Black (33.3%vs. 20.4%, p = 0.04), Hispanic (40%vs. 24.6%, p = 0.03), and on Medicaid (80.6%vs. 31.7%, p < 0.001).

**Table 1:** Patient demographics and characteristics.

Variable	
Biological Sex	
Male	0
Female	36
Age (average, years)	37.5 ± 9.8
BMI (average)	30.1 ± 6.5
Diabetes	1
Tobacco/marijuana use	10
Race	
Black	12
Asian	2
Hispanic	14
Caucasian	7
Insurance	
Commercial	6
Medicaid	29
Medicare	1

Tobacco/cannabis use and obesity were prevalent in 25% and 47.2% of patients, respectively.

### Geographic distribution of index procedures

Most patients underwent aesthetic procedures in the United States (52.8%), followed by Mexico (36.1%) and the Dominican Republic (11.4%). The states in which procedures were conducted in the U.S. include Arizona (5.3%), California (47.4%), Florida (31.6%), and Texas (5.3%).

### Intervention categories

Body regions intervened upon were the abdomen (52.8%), breasts (52.8%), buttocks (33.3%), and arms (8.3%) (Table 2). In 36.1% of patients, multiple body regions were intervened upon during the initial case. Abdominal cases included primary abdominoplasty (84.2%), panniculectomy (10.5%), and revision abdominoplasty (5.3%). Breast cases were primary augmentation (36.8%), revision augmentation (21.1%), augmentation mastopexy (15.8%), mastopexy only (15.8%), and other (10.5%). Buttocks cases were gluteal fat grafting (63.6%) and implant insertion (36.4%). Arm cases were all brachioplasty.

**Table 2:** Index surgery characteristics.

Variable	
Surgery Location	
United States	19
Dominican Republic	4
Mexico	13
Surgery Type	
Abdominoplasty	16
Revision abdominoplasty	1
Panniculectomy	2
Primary Augmentation	7
Revision Augmentation	4
Augmentation mastopexy	3
Mastopexy	3
Breast – other	1
Liposuction	12
Gluteal implants or fat grafting	4
Brachioplasty	3

### Complications

Complications included infection (44.4%), dehiscence (16.7%), pain (16.7%), seroma/drainage (16.7%), drain management (8.3%), and hematoma (2.8%). Abdominoplasties were associated with the highest proportion of complications (47.1%), followed by breast augmentation (35.3%). A detailed list of complications related to specific procedures are enumerated in Table 3. Patients using tobacco/cannabis were more likely to present with infection (88.9%vs. 25.9%, p = 0.001).

**Table 3:** Procedure specific complications.

Index surgery	Complication	Total	U.S.	Non-US
Abdominoplasty	Drain management	2	1	1
	Infection	4	1	3
	Pain	2	0	2
	Seroma/drainage	5	1	4
	Wound dehiscence	3	2	1
Brachioplasty	Hematoma	1	0	1
Breast augmentation	Infection	9	5	4
	Pain	1	1	0
	Wound dehiscence	2	2	0
Breast mastopexy	Wound dehiscence	1	1	0
Breast reduction	Infection	1	1	0
Breast – other	Pain	1	1	0
Gluteal implant	Drain management	1	0	1
	Infection	2	1	1
	Pain	2	2	0
Panniculectomy	Seroma/drainage	1	1	0

Amongst those with infection, the following organisms were identified: *Escherichia coli* (12.5%), *Bacteroides thetaiotaomicron* (6.3%), *Prevotella bivia* (6.3%), *Staphylococcus aureus* (31.3%), *Staphylococcus epidermidis* (12.5%), *Bacteroides fragilis* (6.3%), *Finegoldia magna* (12.5%), *Candida albicans* (6.3%), *Streptococcus agalactiae - group B* (6.3%). Multi-drug resistant organisms were identified in four cases which included extended-spectrum beta-lactamase *E. coli* (6.3%) and *methicillin-resistant Staphylococcus aureus* (18.8%).

### Healthcare utilization and intervention

Median postoperative day was 21 (Interquartile Range [IQR] 11.5, 36). Procedures conducted abroad had a median postoperative day of 19 (IQR 12.27, 28) compared to 25 (IQR 11.5, 59) for procedures performed within the U.S. There were recurrent emergency department visits among 22.2%. Nearly half received computed tomographic imaging (47.2%). Half of patients were admitted with a median length of stay of 2.5 days (IQR 1, 3). One-third underwent intervention, including implant removal (58.3%), image-guided aspiration (25%), and incision and drainage (16.7%). Patients using tobacco/cannabis were more likely to undergo intervention (66.7%vs. 22.2%, p = 0.01).

Overall, 44.4% of patients had outpatient follow-up visits for a median of 3 (IQR 2, 4) visits up to a median of 38 (IQR 21.5, 70.5) days after consultation, with an average follow-up duration of 20.9 ± 44.8 days.

### Fiscal impact

For the original hospital encounter and any subsequent outpatient/emergency visits, the total median hospital charge was $43,324.96 (IQR $10,728.12, $80,803.18) and median insurance payment was $3947 (IQR $404.61, $24,516.00). In the setting of operative intervention, median hospital charge and insurance payments were $125,358.70 (IQR $51,065.52, $152,704.70) and $14,863.52 (IQR $3947, $49,031.13), respectively.

## Discussion

### Socioeconomic disparities and health equity

Our findings that patients were significantly more likely to be Black, Hispanic, and/or on Medicaid insurance highlights important health equity concerns in cosmetic surgery and align with broader literature on health disparities in plastic surgery. Although accounts of fewer minor surgical complications amongst racial/ethnic minorities exist in the literature,^9^ studies overwhelmingly demonstrate that minority racial groups and patients with lower socioeconomic status experience the greatest burden of inequity in plastic surgery, including barriers to treatment access, offers of care, and higher rates of post-surgical complications.^10^, ^11^, ^12^, ^13^ Interestingly, studies have found that even when accounting for economic limitations and controlling for insurance status, Blacks are subject to delays in treatment and suggesting there are systemic factors that must be addressed.^14^

Patients from economically disadvantaged backgrounds may be more likely to seek low-cost procedures without adequate consideration of surgeon qualifications, facility accreditation, or complication risks. This population is not only vulnerable to decreased health literacy, but also vulnerable to insurance churn,^15^ whereby insurance stability is tenuous and fluxes between cycles of having and not having insurance secured. This insurance instability can adversely impact postoperative care, follow up visit attendance, and delays in presentation when complications first arise. As the cosmetic surgery population becomes more diverse, it is increasingly important for surgeons to be cognizant of cultural and socioeconomic factors that may impact surgical decision-making, patient satisfaction, and surgical outcomes.

A recent literature review found that over 80% of studies focus on identifying disparities but fewer than 11% of studies are dedicated to evaluating underlying causes and interventions.^10^ Many of these studies also focus on reconstructive and craniofacial procedures and there is a paucity of literature on disparities in aesthetic surgery. There is a dual need for research that moves beyond detection to actively reducing disparities through evidence-based interventions, policy initiatives, and patient-centered care and attention towards cosmetic procedures.

### Risk factors and complications

The strong association between tobacco/cannabis use and infection (p = 0.001) and need for operative intervention (p = 0.01) underscores the importance of thorough pre-surgical clinical assessment and patient counseling. Smoking is a well-established independent risk factor for postoperative complications in plastic surgery, with effects mediated primarily through impaired oxygen delivery to tissues and compromised wound healing. Large database studies have demonstrated that tobacco users have significantly increased odds of surgical complications, wound complications, and wound dehiscence following plastic surgery procedures.^16^ Given that patients with tobacco use are still being treated in the elective setting despite the known post-surgical sequelae in the medical community, there may either be incomplete patient knowledge or a lax discernment being exercised towards a patient’s surgical readiness. If true, there may be room for a third-party entity to offer firm regulation or risk stratifications for this group.

The high prevalence of obesity in this cohort is also noteworthy, as obesity is another well-known, independent risk factor for surgical site infections regardless of specialty and infections and venous thromboembolism in aesthetic surgery.^17^, ^18^, ^19^ Bigarella et al. found that amongst plastic surgery procedure categories, patients with high BMI undergoing cosmetic procedures were most at risk for complications.^18^ Their finding further emphasizes the need for preoperative risk stratification and safe surgical practices.

### Repercussions of cosmetic tourism

Our findings that nearly half of aesthetic procedures were performed abroad, primarily in Mexico and the Dominican Republic, which are countries commonly reported to be destinations for elective cosmetic procedures.^20^, ^21^, ^22^ Patients may travel for aesthetic procedures due to geographic imbalances in access to care. Regions with high demand but relatively low surgeon supply may lack sufficient local capacity, while areas with greater provider density may offer more competitive pricing, incentivizing patients to seek care elsewhere.^23^^,^^24^ Notably, these patients who travel abroad for cosmetic surgery are often exposed to unique risks beyond those inherent to the procedures themselves.^25^ These include different surgical and infection-control practices and exposure to antibiotic-resistant organisms more prevalent in certain countries.^7^^,^^26^, ^27^, ^28^, ^29^ Our findings suggest that complication profiles are similar between domestically and internationally performed aesthetic operations, consistent with prior literature.^22^^,^^25^ Reported differences may instead relate to variations in postoperative follow-up, as internationally performed procedures are often associated with reduced continuity of care and delayed management of complications.^30^^,^^31^

Regardless, patients’ home countries often face the brunt of managing complications in which original preoperative planning, knowledge of intraoperative findings, or established postoperative protocols may be unknown.^7^^,^^25^ The psychological and professional toll on individual surgeons, while not extensively discussed in the literature, is compounded by managing preventable complications, navigating possible patient dissatisfaction, and addressing aesthetic outcomes without knowledge of the original surgical plan.

### Financial implications

The substantial financial burden documented in this study—with median hospital charges exceeding $43,000 and reaching $125,000 for patients requiring operative intervention—highlights the hidden and unforeseen costs of elective cosmetic surgery. While patients may save money on the initial procedure, complications can reportedly result in costs north of $250,000 for lengthened hospital admissions that far exceed any initial savings.^31^^,^^32^

The large discrepancy between hospital charges and insurance payments (median payment of $3947 versus charges of $43,324.96) reflects the financial burden absorbed by healthcare institutions. For Medicaid patients, this gap is particularly pronounced given Medicaid's significantly lower reimbursement rates compared to Medicare and commercial insurance.^9^ This creates a substantial unfunded burden on academic medical centers and safety-net hospitals that provide care for these patients.

Previous cost analyses have similarly demonstrated the financial impact of cosmetic surgery tourism complications on public healthcare systems in other countries as well. UK studies reported costs up to £42,083.59 perhaps partially contributing to the British Associate of Aesthetic Plastic Surgeons heavy caution against individuals’ traveling abroad for surgery.^32^, ^33^, ^34^ Ultimately, these costs represent not only direct medical expenses but also the opportunity cost of resources diverted from other patients and services.

### Limitations

The retrospective design and single-center experience may limit generalizability. The sample size of 36 patients, while comparable to other single-institution studies, may be underpowered to detect certain associations. Selection bias may exist, as patients presenting to an academic medical center may differ from those seeking care elsewhere or not seeking care at all. Financial data reflect hospital charges and insurance payments but do not capture indirect costs such as lost productivity, long-term disability, or psychological impact. Additionally, while appropriate follow up was conducted for patients, standardized outcome data and questionnaires were not obtained for this study and may be an area for future research. Finally, we did not have access to detailed information about the original procedures, surgeon qualifications, or facility characteristics, which limits our ability to identify specific risk factors related to the initial surgery.

## Conclusion

This study demonstrates a disproportionate number of patients from socioeconomically disadvantaged backgrounds—particularly Black and Hispanic patients and those with Medicaid insurance—afflicted with complications from elective aesthetic surgery procedures. As the cosmetic surgery population becomes increasingly diverse,^35^ our findings highlight important health equity concerns and underscore the need for enhanced patient education, preoperative risk stratification, regulatory oversight, and targeted interventions to reduce disparities in cosmetic surgery access and outcomes.

Additionally, costs associated with managing these complications fall disproportionately on patients and healthcare systems. Addressing this issue can not only optimize patient care and quality of life, but it can also generate a more robust healthcare system that can promote meaningful resource allocation.

## Funding

None.

## Ethical approval statement

Approval for this study was obtained by the University of California, Davis institutional review board (IRB study #1593453-2).

## Declaration of competing interest

None.

## Appendix Supplementary materials

Image, application 1
